# Fluorescent PLGA Nanocarriers for Pulmonary Administration: Influence of the Surface Charge

**DOI:** 10.3390/pharmaceutics14071447

**Published:** 2022-07-11

**Authors:** Aina Areny-Balagueró, Wid Mekseriwattana, Marta Camprubí-Rimblas, Andrea Stephany, Ariana Roldan, Anna Solé-Porta, Antonio Artigas, Daniel Closa, Anna Roig

**Affiliations:** 1 Institut d’Investigació i Innovació Parc Taulí (I3PT), 08208 Sabadell, Spain; aareny@tauli.cat (A.A.-B.); mcamprubi@tauli.cat (M.C.-R.); 2 Faculty of Medicine, Universitat Autònoma de Barcelona, 08193 Bellaterra, Spain; 3 Institut de Ciència de Materials de Barcelona, ICMAB-CSIC, Campus UAB, 08193 Bellaterra, Spain; widd.mek@gmail.com (W.M.); andreastephany26@gmail.com (A.S.); ariana.roldan.carretero@gmail.com (A.R.); asole@icmab.es (A.S.-P.); 4 School of Materials Science and Innovation, Faculty of Science, Mahidol University, Bangkok 10400, Thailand; 5 Centro de Investigaciones Biomédicas en Red de Enfermedades Respiratorias, CIBERES-Instituto De Salud Carlos III, 28029 Madrid, Spain; 6 Servei de Medicina Intensiva, Corporació Sanitària i Universitària Parc Taulí, 08208 Sabadell, Spain; 7 Institut d’Investigacions Biomèdiques de Barcelona, Consejo Superior de Investigaciones Científicas (IIBB-CSIC), 08036 Barcelona, Spain

**Keywords:** nanocarriers, lung, PLGA, surface charge, intratracheal instillation, cell uptake

## Abstract

Nearly four million yearly deaths can be attributed to respiratory diseases, prompting a huge worldwide health emergency. Additionally, the COVID-19 pandemic’s death toll has surpassed six million, significantly increasing respiratory disease morbidity and mortality rates. Despite recent advances, it is still challenging for many drugs to be homogeneously distributed throughout the lungs, and specifically to reach the lower respiratory tract with an accurate sustained dose and minimal systemic side effects. Engineered nanocarriers can provide increased therapeutic efficacy while lessening potential biochemical adverse reactions. Poly(lactic-co-glycolic acid) (PLGA), a biodegradable polymer, has attracted significant interest as an inhalable drug delivery system. However, the influence of the nanocarrier surface charge and its intratracheal instillation has not been addressed so far. In this study, we fabricated red fluorescent PLGA nanocapsules (NCs)—Cy5/PLGA—with either positive (Cy5/PLGA+) or negative surface charge (Cy5/PLGA-). We report here on their excellent colloidal stability in culture and biological media, and after cryo-storage. Their lack of cytotoxicity in two relevant lung cell types, even for concentrations as high as 10 mg/mL, is also reported. More importantly, differences in the NCs’ cell uptake rates and internalization capacity were identified. The uptake of the anionic system was faster and in much higher amounts—10-fold and 2.5-fold in macrophages and epithelial alveolar cells, respectively. The in vivo study demonstrated that anionic PLGA NCs were retained in all lung lobules after 1 h of being intratracheally instilled, and were found to accumulate in lung macrophages after 24 h, making those nanocarriers especially suitable as a pulmonary immunomodulatory delivery system with a marked translational character.

## 1. Introduction

The incidence of respiratory diseases is increasing, principally among children, the elderly, and people with enfeebled immune systems. Almost 4 million deaths can be attributed to respiratory diseases yearly, causing a huge worldwide health burden [1,2]. Moreover, the SARS-CoV-2 pandemic, associated with severe pulmonary syndromes in most patients, has led to more than 6 million deaths (according to the WHO Health Emergency Dashboard), significantly increasing respiratory-associated diseases’ morbidity and mortality rates [3]. Even after recent advances, a decrease in systemic effects while at the same time achieving an effective dose and a homogeneous drug distribution in the lungs—including the lower respiratory tract—is still challenging for certain drugs [4].

The past two decades have witnessed a considerable innovation leap in the drug delivery field with the use of engineered nanocarriers, as they allow an increase in therapeutic efficacy while lessening potential biochemical adverse reactions [5]. It should be noted that the encapsulation of the pharmaceutical compounds can also have beneficial effects in terms of higher drug stability, the possibility to co-deliver various active components, the enhancement of specific interactions with the target organs, and more limited drug accumulations in healthy tissues [6]. However, to attain improved functionality, lack of cytotoxicity, and biocompatibility of the NCs, precise control of the carrier composition, media dispersibility, surface charge, size, size distribution, and shape is needed [7]. Consequently, a thorough evaluation of pharmacokinetics and pharmacodynamics is required during the development stages of any novel nanoparticle (NP) intended as a drug carrier. In the context of respiratory diseases, hepatic first-passage metabolism, blood clearance, respiratory anatomy, and particle size are key factors affecting the NPs delivery, deposition, and efficacy [8].

NCs made of poly(lactic-co-glycolic acid) (PLGA)—a biodegradable polymer approved by the U.S. Food and Drug Administration (FDA) and the European Medicines Agency (EMA)—have attracted significant interest as an inhalable drug delivery system [9]. The chemical characteristics of the PLGA NCs allow for the modification of their degradation and the drug release profiles [10], the incorporation of imaging moieties, or the addition of organic groups to modify the NCs’ surface [11,12]. It is well established that specific properties such as the charge and size of the NCs have considerable effects on their lung clearance kinetics and retention patterns [13], making PLGA NCs potentially better suited as pulmonary drug delivery systems than lipid NPs—another explored inhalable delivery system [14,15]. Furthermore, preliminary in vivo studies indicate that biodegradable PLGA NPs trigger significantly lower inflammatory responses than non-biodegradable polystyrene NPs [16].

Beyond particle-related factors, the route of administration and the extent of tissue distribution are essential parameters to be considered. Since a lung injury can also be attributable to extrapulmonary causes [17], intravenous administration is sometimes a suitable option, as it facilitates the administration of high doses and allows the drug to reach peripheral organs. However, the intravenous route could become unsuccessful in effectively delivering the therapeutic agents to the lungs at an adequate concentration, and could even trigger unwanted side effects [18]. Consequently, pulmonary administration routes are gaining much importance in addressing respiratory diseases. Inhalation and intratracheal instillation allow for an increased and homogeneous pulmonary dosage, better tissue deposition, and reduced drug levels in the non-target organs, enabling higher efficiency of the administered treatment. Moreover, intratracheal instillation is the most used method in preclinical research. It has frequently been used to assess pulmonary absorption, especially for determining the precise dosing and effectiveness. It is a simpler, less expensive, and more reproducible route than inhalation [19]. Still, regarding translation to the clinic, the inhalation of drugs offers a non-invasive method that enhances patient acceptability and lung concentration [20].

Here, we report the production and the characterization of PLGA nanocarriers developed to improve lung biodistribution and minimize the systemic side effects of pulmonary-administered drugs. Two types of PLGA NCs with either positive or negative surface charge endowed with a red fluorescent tag (cyanine 5) were investigated. We demonstrated the suitability of these new nanocarriers for in vitro and in vivo studies by analyzing their colloidal stability in cell culture media, in biological media, and after cryo-storage. In addition, we evaluated the cytotoxicity of the NCs and their ability to be internalized by alveolar epithelial cells or macrophages, as well as their in vivo biodistribution.

## 2. Materials and Methods

### 2.1. Materials

#### 2.1.1. Suppliers and Purity of the Chemical Reagents

PLGA (lactide:glycolide 50:50, M_w_ 7000–17,000), dicyclohexylcarbodiimide (DCC, 99%), *N*-hydroxysuccinimide (NHS, 98%), ethylenediamine (≥99.5%), PVA (87–90% hydrolyzed, MW 3000–7000), and triethylamine (≥99.5%) were obtained from Sigma-Aldrich. Cyanine5-NHS ester (Cy5, 95%) was obtained from Lumiprobe, and trehalose (D(+)-trehalose 2-hydrate) from AppliChem Panreac.

#### 2.1.2. Synthesis of Amine-Functionalized PLGA (PLGA-NH_2_)

The commercial PLGA was modified through amide formation of PLGA with ethylenediamine, using DCC and NHS as coupling agents [11]. PLGA (300 mg) was dissolved in 8 mL of dichloromethane (DCM) under magnetic stirring. A solution of DCC (20.6 mg, 0.1 mmol) in 1 mL of DCM and NHS (11.5 mg, 0.1 mmol) in 0.5 mL of acetone was added to the polymer solution, and the mixture was left stirring for 4 h at room temperature (RT) to activate the PLGA. To complete the coupling reaction, a solution of ethylenediamine (10 µL, 0.15 mmol) in 1 mL of DCM was added dropwise into the activated PLGA solution, and the reaction was kept under stirring for 2 h. Upon completion, the solution was centrifuged at 9000 rpm for 15 min at 4 °C. The pellets containing dicyclohexylurea byproducts were discarded, and the supernatant was collected and filtered with a 0.2 µm PTFE syringe filter to remove residual precipitates. The resulting PLGA-NH_2_ was obtained through precipitation by slowly adding the supernatant to a 10-fold excess volume of ethanol. PLGA-NH_2_ pellets were collected by centrifugation and redissolved in 3 mL of DCM. Precipitation in ethanol was repeated once, and the purified PLGA-NH_2_ was collected and dried under vacuum.

#### 2.1.3. Synthesis of Fluorescent PLGA (PLGA-Cy5)

PLGA-NH_2_ (100 mg) was mixed with Cy5 (7 mg, 0.01 mmol) and dissolved in 4 mL of acetone. The solution was put under magnetic stirring, and a solution of triethylamine (2.5 mg, 0.025 mmol) in 0.6 mL of acetone was added dropwise to the reaction as a catalyst. The reaction was left stirring for 6 h under dark conditions. Next, the reaction was added to an excess amount of ethanol to precipitate the PLGA-Cy5, followed by centrifugation and drying under vacuum.

#### 2.1.4. Formulation of Fluorescent PLGA NCs with Different Surface Charges

PLGA NCs were formulated using a double-microemulsion solvent evaporation method, as previously reported in [11,12], with minor modifications. Briefly, 50 µL of Milli-Q water as an inner aqueous phase (W_1_) was emulsified in the organic phase (O), which consisted of 50 mg of different proportions of pristine or functionalized PLGA dissolved in 500 µL of DCM. To obtain positively surface-charged NCs (Cy5/PLGA+), the formulation was carried out by mixing PLGA-Cy5 with PLGA-NH_2_ at a 1:1 ratio. Meanwhile, for negatively surface-charged NCs (Cy5/PLGA-), the formulation was carried out by mixing PLGA-Cy5 with pristine PLGA at a 1:1 ratio. Emulsification was performed by sonication at 200 W for 28 s to obtain the first emulsion (W_1_/O). Then, 2 mL of 2% *w/v* PVA solution was added and sonicated for another 28 s to form a second emulsion (W_1_/O/W_2_). The whole sonication process was performed in an ice bath to maintain the temperature at 4 °C. The obtained double emulsion was added to 50 mL of Milli-Q water and mechanically stirred for 2 h at RT to evaporate the organic solvent. The resulting NCs were centrifuged at 900 rpm for 15 min at 4 °C, and the pellets containing large particles were discarded. The supernatant was collected and centrifuged at 9000 rpm for 15 min to separate the NCs. This step was repeated twice. The final NCs were dispersed in 6 mL of trehalose solution (2 mg/mL). The NCs were lyophilized (LYOQUEST-85 from Telstar) and stored at −80 °C for further use.

#### 2.1.5. Characterization of PLGA NCs

A field-emitting scanning electron microscope (SEM, FEI Quanta 200 FEG) and transmission electron microscope (TEM, JEM-1210, JEOL Ltd., Akishima, Tokyo, Japan) were used to study the morphologies of the NCs. For the sample preparation, 0.5 mg (analytical balance, Acculab Atilon ATL-244-) of lyophilized NC powder was redispersed into 1 mL of Milli-Q water and centrifuged at 4000 rpm for 10 min to remove the trehalose. Then, the pellet of NCs was redispersed in 1 mL of fresh water. Finally, 6 μL of the slightly turbid suspension was deposited onto a small piece of a silicon wafer stuck on the top of a carbon layer and dried at room temperature overnight. The sample was then sputtered with 60/40 Au/Pd (20 mA 2 min for 10 nm deposition, Emitech K550, Quorum Technologies Ltd., Lewes, UK). Secondary electron images were taken using a working distance of 8 mm, a large field detector, an acceleration voltage of 5 kV, and a pressure of 60 Pa. TEM samples were prepared by placing and drying one drop of the previously prepared NC dispersion on a copper grid.

The hydrodynamic diameter (D_H_) and zeta potential (ζ) of the NCs were measured using a Zetasizer Nano ZS (Malvern Instruments, Malvern, UK). The samples were prepared by dispersing lyophilized NCs in Milli-Q water to obtain a dispersion with a final concentration of 0.2 mg/mL. The mean hydrodynamic diameter measured by dynamic light scattering (DLS) is a value that refers to how a particle diffuses within a fluid. The diameter that DLS obtains is the diameter of a sphere that has the same translational diffusion coefficient as the particle. The hydrodynamic radius includes the solvent molecules dragged and moved together with the nanocapsules.

The colloidal stability of empty Cy5/PLGA+ and Cy5/PLGA- NCs in different media was determined by analyzing the NCs’ size as a function of time (n = 3). For that, 1 mg of NCs was dispersed in 1 mL of water, saline (0.9% NaCl), RPMI-1640, and DCCM-1 culture media (Biological Industries, Kibbutz Beit HaEmek, Israel), or in the bronchoalveolar lavage supernatant (SNBAL) of rat lungs and sera. A final volume of 1.2 mL was used for each sample. The mean D_H_ of the NCs in each medium was then measured at t = 0, 6, 12, 24, and 48 h. Before each measurement, the solutions were agitated on a Thermo-Shaker (Biosan, TS-100, Bunkyo, Japan) at 37 °C and 400 rpm. Fluorescence spectra were taken, and the results showed that Cy5/PLGA- has approximately 1.4 times higher emission intensity than its positively charged counterpart.

#### 2.1.6. Cell Harvesting

Alveolar type II (ATII) cells: Fresh ATII cells were isolated from healthy male donor Sprague-Dawley rats (225–250 g). Briefly, the lungs were removed from each animal and subjected to bronchoalveolar lavage (BAL) 5 times with 10 mL of saline. The lungs were digested with 0.25% trypsin (T8003; Sigma, St. Louis, MO, USA) and kept submerged in a saline bath at 37 °C for 30 min. After digestion, fetal bovine serum (FBS; Gibco^TM^, Thermo Fisher Scientific, Waltham, MA, USA) was added to stop trypsin activity, and then the lungs were chopped into 1–2 mm^3^ cubes, treated with DNase I (75 U/mL) (Roche Diagnostics, Manheim, Germany), and filtered through nylon meshes (pore size 40–100 µm). The resulting cell suspension was centrifuged without brake or acceleration (500× *g*, 20 min) through a sterile Percoll gradient, and the ATII-cell-rich band was collected. A second DNase treatment was administered, and the cells were recovered as a pellet by centrifugation at 500× *g* for 15 min. These cells were suspended in DCCM-1 supplemented with 10% FBS, 2 mM L-glutamine, 100 U/mL penicillin, and 100 µg/mL streptomycin, and subjected to differential attachment on a plastic Petri dish. Non-adherent ATII cells were collected after 1 h, and were counted to establish the final yield of freshly purified cells. ATII cells’ viability was assessed with trypan blue (Sigma, St. Louis, MO, USA), and their purity was assessed by alkaline phosphatase staining (Sigma, St. Louis, MO, USA). The expression of surfactant C (SPC, Santa Cruz, Dallas, TX, USA, ref sc-13979, rabbit, 1:100) was measured by immunofluorescence and marked by the secondary anti-rabbit antibody (Santa Cruz, 136 USA, ref. sc2359. FITC, 1:100).

THP-1: Human monocytic cell line derived from an acute monocytic leukemia patient (American Type Culture Collection). Human THP-1 cells were cultured in suspension in RPMI 1640 medium supplemented with 10% FBS, 2 mM L-glutamine, 100 U/mL penicillin, and 100 µg/mL streptomycin. Cells were differentiated to macrophages through a first incubation with 50 nM phorbol 12-myristate 13-acetate (PMA) (Sigma-Aldrich) for 24 h. After that, the PMA-containing medium was replaced with fresh medium without PMA and further incubated for 24 h.

HPAEpiC: Human pulmonary alveolar epithelial cells (HPAEpiC), also called human pneumocytes, were separated from human lung tissue (ScienCell Research Laboratories, Innoprot, USA). This cell line comprises 2 subtypes of epithelial cell: type I alveolar cells (ATI) and type II alveolar cells (ATII). HPAEpiC cells were cultured with Alveolar Epithelial Cell Medium (AEpiCM, ScienCell Research Laboratories, Carlsbad, CA, USA; Innoprot, Bizkaia, Spain) with 2% FBS, 1% EpiGS, and 100 µg/mL streptomycin in previously poly-L-lysine-coated cell culture flasks.

#### 2.1.7. In Vitro MTS Cytotoxicity

The potential cytotoxic effect of Cy5/PLGA+ and Cy5/PLGA- NCs on ATII and THP-1 cells was analyzed by MTS assay (Promega, Wisconsin, USA) (n = 3). This assay is based on the ability of live cells to reduce a tetrazolium salt—MTS (3-(4,5-dimethylthiazol-2-yl)-5-(3-carboxymethoxyphenyl)-2-(4-sulfophenyl)-2H tetrazolium)—to purple formazan in the presence of phenazine methosulfate. ATII and THP-1 cells (7 × 10^4^ and 5 × 10^3^ cells per well, respectively) were seeded in 96-well plates and allowed to attach for 24 h. Cells were then treated with increasing concentrations of NCs (1, 3, 6, and 10 mg/mL) at 37 °C. After treatment, cell viability was determined at 24 and 48 h for ATII cells, and at 24 h for THP-1 cells. The amount of formazan generated was quantified by measuring the absorbance at 490 nm on a microplate reader.

#### 2.1.8. Confocal Analysis for Cy5/PLGA- and Cy5/PLGA+ Cellular Uptake In Vitro

HPAEpiC cells and differentiated THP-1 cells with a density of 5 × 10^5^ cells/well were seeded in a 48-well plate with sterilized coverslips. Then, 1 mg/mL of Cy5/PLGA- and Cy5/PLGA+ NCs was added to the cells for 0.5, 1, 2, and 4 h for THP-1 cells, and for 8, 24, 48, and 72 h in the case of HPAEpiC cells. The excess NCs were washed with PBS at each time point, and the cells were fixed with 4% formaldehyde. The plasma membrane of the cells was stained with PKH67 green fluorescent cell-linker dye (Sigma-Aldrich, St. Louis, MO, USA), stopping the reaction with FBS. Cell nuclei were dyed with the DNA-specific blue fluorescent stain 4,6-diamidino-2-phenylindole (DAPI) (Sigma-Aldrich, St. Louis, MO, USA). Coverslips were fixed, and the stained cells were mounted with Fluoromount Aqueous Mounting Medium (Sigma, St Louis, MO, USA). An evaluation was performed using an inverted Nikon Eclipse Ti2-E microscope (Nikon Instruments, Melville, NY, USA) attached to the Andor Dragonfly spinning disk unit. Samples were excited with 405 nm, 488 nm, and 649 nm laser diodes. Cells were imaged on a high-resolution scientific complementary metal–oxide–semiconductor (sCMOS) camera (Zyla 4.2, 2.0 Andor, Oxford Instruments Company, Concord, MA, USA), and Z-stacking was recorded at a 0.15 µm interplanar distance. Fusion software from Oxford Instruments Company was used for the acquisition of images. Image deconvolution was performed after acquisition. Ten samples’ images were processed (with an average of 15 cells in each; n = 2) and analyzed with ImageJ/Fiji open-source software using a customized ImageJ macro.

#### 2.1.9. Animals

Male Sprague-Dawley rats (Charles River, Écully, France) weighing 200–225 g were used in accordance with the European Community Directive 86/609/EEC and the Spanish guidelines for experimental animals. The experimental protocol was approved by the Animal Research Ethics Committee of the Autonomous University of Barcelona (UAB) and the Animal Experimentation Committee of the Generalitat de Catalunya, with the animal studies approval number 11220.

#### 2.1.10. In Vivo Lung Biodistribution and Cellular Uptake of NCs

Cy5/PLGA- NCs at a concentration of 6 mg/mL, diluted in saline to a total volume of 500 µL, were administered via two different routes: intravenously (tail vein), or intratracheally instilled (n = 1) for 1 h for lung biodistribution studies. To determine the in vivo cellular uptake of the NCs, the same volume and concentration of Cy5/PLGA- NCs were intratracheally instilled for 24 h (n = 3). At the end of treatment, all animals were anesthetized intraperitoneally with ketamine (90 mg/kg)/xylazine (10 mg/kg) (3:1), and were euthanatized by exsanguination of the abdominal aorta.

NCs’ in vivo biodistribution was analyzed by histological studies. Multilobular lung (each lobule separately) and liver were removed, embedded in Tissue-Tek^TM^CRYO-OCT (Science Services), and processed in 14 μm sections with the cryotome. Plasma membranes of the histological slices were stained with a green Cell Mask (Thermo Fisher Scientific) and mounted with Fluoromount Aqueous Mounting Medium (Sigma, St. Louis, MO, USA). Four slices from each sample were analyzed using a confocal microscope (Inverted microscope Leica DMI 4000B), and Z-stacking was recorded at a 0.15 µm interplanar distance. Green and red fluorescence were obtained using 488 nm and 649 nm laser excitation, respectively, 60× magnification, and an average zoom = 3. Images were processed using the software platform of LAS X Life Science (Leica Microsystems, Wetzlar, Germany) combined with ImageJ (X64, v. 2.1.4) software.

In vivo cellular uptake of NCs was studied by flow cytometry analysis. Unilobular lung was removed and perfused with a constant flow of 25 mL of 0.25% trypsin solution for 15 min. After that, FBS was added to stop trypsin activity, and then the lungs were chopped into 1–2 mm^3^ cubes, treated with DNase I (75 U/mL), and filtered through nylon meshes (pore size 40–100 µm). Then, 2 mL of the resulting cell suspension was centrifuged for 5 min at 500× *g*, and the pellet was treated with ammonium chloride potassium (ACK) buffer to lysate the erythrocytes. After washing and Fc blocking with CD16/CD32 antibody, the cells were stained with the antibody mix at 4 °C in the dark (Table 1). After 30 min of incubation, cells were washed and measured using a FACSCanto II cytometer for different cell leukocyte subsets’ counts, measurement, and classification. Data were analyzed using FlowJo.

Cell subset populations were gated as follows after selecting singlets—total myeloid cells: CD45+ and CD11b+; monocytes: CD45+ CD11b+, and His48+; ATII cells: CD45+, CD11b-, and AP+ (alkaline phosphatase). ATII cells and monocytes that were positive for Cy5 fluorescence were counted.

#### 2.1.11. Statistical Analysis

The data were analyzed using GraphPad Prism 7 software (Domatics, San Diego, CA, USA)and expressed as the mean ± standard error of the mean (SEM). One-way ANOVA with the Newman–Keuls multiple comparison test was applied to compare more than two groups, and two-way ANOVA followed by Bonferroni’s multiple comparison test was used to analyze data with more than one variable. All statistical tests conducted were two-sided, and *p* < 0.05 was considered significant.

## 3. Results and Discussion

### 3.1. Synthesis of PLGA Nanocapsules

**Figure 1:**
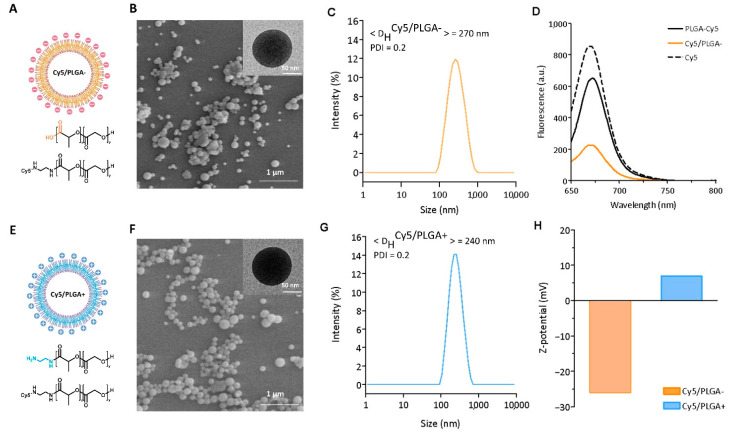
Representative schemes (created with Biorender.com), SEM images, and hydrodynamic diameter distribution of Cy5/PLGA- (**A**–**C**) and Cy5/PLGA+ (**E**–**G**) NCs. TEM image of a single NC (insets of (**B**,**F**)). Fluorescence spectra comparison of free Cy5, PLGA-Cy5, and Cy5/PLGA- NCs. Measurements were performed under an excitation wavelength of 650 nm (**D**). Zeta-potential (ζ) of the Cy5/PLGA- and Cy5/PLGA+ NCs (**H**).

The NCs were synthesized with negative and positive surface charges using either commercial PLGA with carboxylic terminal groups or PLGA-NH_2_. To obtain PLGA-NH_2_, the carboxylic groups were activated with NHS and DCC, and reacted with ethylenediamine to further yield PLGA-Cy5. The Cy5/PLGA- NCs were synthesized using a 1:1 mixture of pristine PLGA with PLGA-Cy5, while the cationic Cy5/PLGA+ NCs were obtained by substituting pristine PLGA with PLGA-NH_2_. The schematic representation of the NCs and the chemical structures of the compositions (Cy5/PLGA- NCs and Cy5/PLGA+ NCs) are shown in Figure 1A,E. The different surface charges were confirmed by measuring the ζ values, as shown in Figure 1H. The Cy5/PLGA- showed ζ = −26 mV, while its positively charged counterpart had ζ = 7 mV. Both NCs were spherical with smooth surfaces, as seen in the SEM images (Figure 1B,F). The mean diameter was circa 200 nm for both samples as measured from the TEM images (insets), while the D_H_ values were slightly larger in both systems, at approximately 250 nm, with narrow polydispersity (polydispersity index ≈ 0.2) (Figure 1C,G). As mentioned, Cy5 was incorporated into the NCs for fluorescent tracking purposes, and the dye’s presence was confirmed by fluorescence spectroscopy. The NCs exhibited fluorescent emission at 665 nm when excited at 650 nm, matching that of free Cy5 and the PLGA-Cy5 used as the precursor for the NCs (Figure 1D).

### 3.2. Colloidal Stability of the PLGA Nanocapsules in Culture and Biological Media

**Figure 2:**
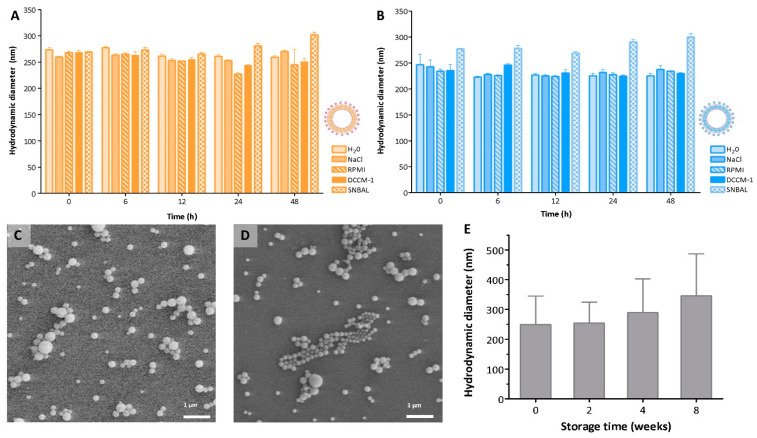
Hydrodynamic diameter of Cy5/PLGA- (orange) and Cy5/PLGA+ (blue) in different biologically relevant media (**A**,**B**). SEM images of Cy5/PLGA- as-synthesized and after storage under ultrafreezing conditions for 8 weeks (**C**,**D**). Hydrodynamic diameter of Cy5/PLGA- after storage under ultrafreezing conditions at different time points (**E**). Data are representative of 3 independent experiments (mean ± SEM). ANOVA followed by a Newman–Keuls multiple comparison test was used.

Lack of colloidal stability in biological environments could hinder the final development of a nanomedicine platform. The formation of aggregates has been shown to alter the behavior of the NPs and have an impact on their cellular uptake [21,22], biodistribution [23], or toxicity [24]. NPs’ aggregation and flocculation in complex biological media is mainly due to the presence of ionic salts and biomolecules, which can adsorb on the NPs’ surface and induce clustering [25]. Particle aggregation can be overcome by introducing either steric hindrance or electrostatic repulsion between the NPs. The colloidal stability of our electrostatically stabilized NCs was evaluated by measuring the D_H_ values in saline, RPMI-1640, and DCCM-1 cell culture media and biological SNBAL (Figure 2A,B). Upon dispersion, both Cy5/PLGA- and Cy5/PLGA+ showed no significant aggregation for up to 48 h, as the D_H_ values were comparable to those found in water (*p* > 0.05). Both Cy5/PLGA- and Cy5/PLGA+ contained charged surfaces, as evidenced by the zeta potential values (Figure 1H), where the charge density was enough to prevent electrostatic screening; thus, our NCs were stable even in saline solution. However, when dispersed in serum, the NCs showed different colloidal behavior (data not shown). The high protein content of serum could interact with the NCs, changing their polydispersity and size distribution [26]. This result suggests that intravenous administration might not be the most favorable route for these NCs. However, overall, the NCs showed excellent colloidal stability in most media tested, and were suitable for further in vitro and in vivo studies.

Moreover, the NCs can be easily lyophilized using trehalose as a cryoprotectant and stored at −80 °C before usage. Figure 2C,D show SEM images of as-synthesized Cy5/PLGA- NCs before and after lyophilization and storage for 8 weeks, along with the same for Cy5/PLGA+ NCs (SEM images not shown). Both samples maintained their shape and size compared to the as-synthesized material. Lyophilized Cy5/PLGA- and Cy5/PLGA+ NCs were resuspended in water, and D_H_ was measured after 2, 4, and 8 weeks of storage. Only after 8 weeks of cryo-storage was there a slight increase in the mean diameter (not statistically significant, *p* > 0.05). These results suggest that a certain degree of irreversible aggregation occurred with lyophilization, indicating a recommended use for the NCs within 4 weeks after their production.

### 3.3. Nanocapsules’ Cytotoxicity

**Figure 3:**
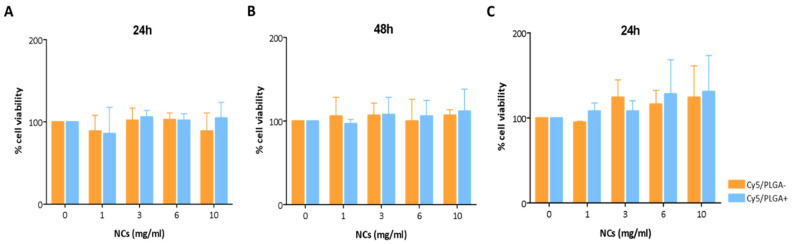
Cell viability of ATII cells treated with the NCs at different concentrations for 24 and 48 h (**A**,**B**). Cell viability of THP-1 cells treated with the NCs for 24 h (**C**). The experiments were performed by MTS assay. Data were normalized to the control (untreated) cell viability (100%) and expressed as the percentage of the mean ± SEM of three independent experiments performed in triplicate. ANOVA followed by a Newman–Keuls multiple comparison test was used (no differences between the control and NC-treated groups).

The cytotoxicity of the NCs to ATII and THP-1 cells was evaluated using an MTS assay. These cell types were selected because macrophages and alveolar epithelial cells are essential in maintaining lung homeostasis and protecting the organism against infection [27]. Macrophages are the principal immune cells in the lung. Their function mainly regulates inflammatory responses through their phagocytic and secretory activity, facilitating the removal of harmful pathogens and simultaneously promoting tissue repair by inducing the proliferation and differentiation of lung-resident epithelial cells [28]. These also contribute to lung biomechanics and local immune response by modulating macrophage functions [27]. The crosstalk between lung-resident cells is an essential aspect to be considered when developing new therapeutic strategies, since it is necessary for maintaining the lungs’ immune balance and response to pathogens. Figure 3A,B show the effects of Cy5/PLGA- and Cy5/PLGA+ on ATII cell viability at 24 and 48 h, respectively. Neither system showed any effect on cell viability, even for concentrations as high as 10 mg/mL. In the case of THP-1 cells, NCs also showed no cytotoxicity after 24 h of exposure, although a minor increase in cell viability was observed (not statistically significant; *p* > 0.05) (Figure 3C). It is well established that macrophages play an essential role in the immune response, and can polarize, reacting to environmental alterations [29]. Indeed, an external stimulus can change the mitochondrial metabolism and physiology underlying the state of macrophage activation [30], leading us to hypothesize that the slightly increased viability of THP-1 is due to the activation of their mitochondrial metabolism in response to the NCs, which would explain the higher amount of formazan product in the MTS assay.

### 3.4. Nanocapsules’ Cellular Uptake

Although it has been reported that surface charge remarkably influences the cellular entry and final intracellular localization of NPs, it is well established that this aspect also strongly depends on the cell type [31]. The cellular uptake efficiency of Cy5/PLGA- and Cy5/PLGA+ was evaluated in THP-1 and HPAEpiC cells due to their relevance and implications for respiratory diseases [32]. As mentioned above, the crosstalk between lung resident cells could be advantageous in developing new therapeutic strategies, since it is essential to maintain the lungs’ immune balance and respond to pathogens [26].

**Figure 4:**
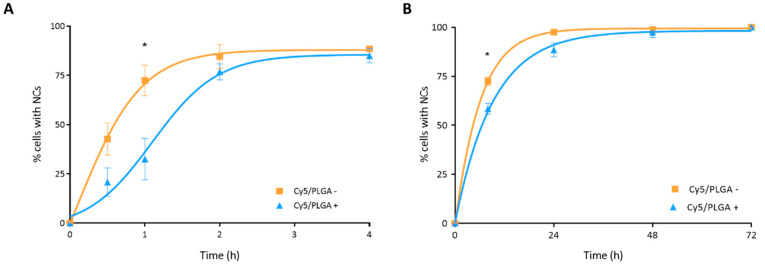
Representation of THP-1 and HPAEpiC cells’ internalization kinetics of PLGA NCs: Circa 90% of macrophages have phagocytized both Cy5/PLGA- and Cy5/PLGA+ after 4 h of treatment; however, the Cy5/PLGA- system shows a faster uptake at the beginning of the treatment (**A**). All (100%) of epithelial cells uptake both Cy5/PLGA- and Cy5/PLGA+ NCs after 72 h of treatment; however, the Cy5/PLGA– system shows a faster uptake at 8 h of treatment (**B**). Data are representative of 2 independent experiments (mean ± SEM); two-way ANOVA followed by Bonferroni’s multiple comparison test was used to evaluate significant differences: * *p* < 0.01 vs. 1 h Cy5/PLGA+ (**A**); * *p* < 0.001 vs. 8 h Cy5/PLGA+ (**B**).

Cellular uptake of NCs was analyzed by detecting the red fluorescence of NCs via confocal microscopy. In the case of THP-1 cells, almost 90% of macrophages showed uptake of NCs after 4 h of treatment (Figure 4A). On the other hand, although both types of NCs showed very similar absorption kinetics, uptake was significantly faster for the Cy5/PLGA– system at 1 h of treatment (*p* < 0.01). This result contrasts with previous studies, showing that NPs with a positive surface charge show better cell uptake than those with a negative surface charge. This might be related to the ζ = 7 mV for the Cy5/PLGA+ NCs, which is lower than the zeta-potential values of the NCs used in the previously cited studies (>30 mV) [24,33,34].

HPAEpiC cells showed slower NC kinetics of internalization compared to THP-1 cells. After 4 h of treatment, epithelial cells showed no detectable red fluorescence (data not shown). It took 24 h of treatment for HPAEpiC cells to reach internalization levels similar to those seen in THP-1 cells after 4 h of treatment. These results are consistent with those of previous studies showing faster cellular uptake in phagocytic cells [24,35]. Moreover, Kuhn et al. demonstrated that macrophages and epithelial cells might take up the NCs through different mechanisms, which could explain the different internalization rates [36]. Moreover, as observed in THP-1 cells, both types of NCs showed similar internalization kinetics by HPAEpiC cells, reaching 100% uptake at 72 h, even though the Cy5/PLGA- NCs exhibited a significantly enhanced uptake in the early stages (*p* < 0.001) (Figure 4B).

**Figure 5:**
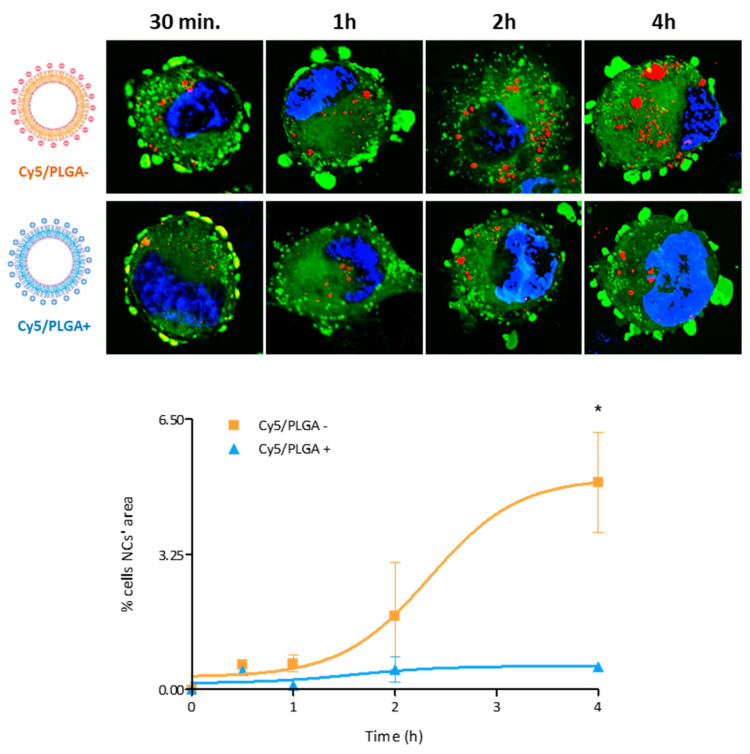
Confocal images of intracellular localization of NCs in THP-1 cells: Representation of the percentage of the cellular area that PLGA NCs occupy. Cy5/PLGA- NCs increased their deposition within the cells in a time-dependent manner (reaching a final occupancy of 5% of the total cell area), while the area occupied by Cy5/PLGA+ NCs remained stable (not exceeding 0.6% of the total cell area). Nuclei are blue (DAPI), cells’ cytoplasm is green (Cell Mask), and NCs are red (Cy5); 63× magnification. Data are representative of 2 independent experiments (mean ± SEM); two-way ANOVA followed by Bonferroni’s multiple comparison test was used to evaluate significant differences; * *p* < 0.001 vs. 4 h Cy5/PLGA+.

Regarding the intracellular distribution of the NCs, in THP-1 cells, Cy5/PLGA- and Cy5/PLGA+ showed very different patterns. As depicted in Figure 5, while the Cy5/PLGA- NCs gradually increased their intracellular accumulation, reaching a final occupancy of circa 5% of the total cell area, the Cy5/PLGA+ NCs did not exceed 0.6% cell surface occupancy.

**Figure 6:**
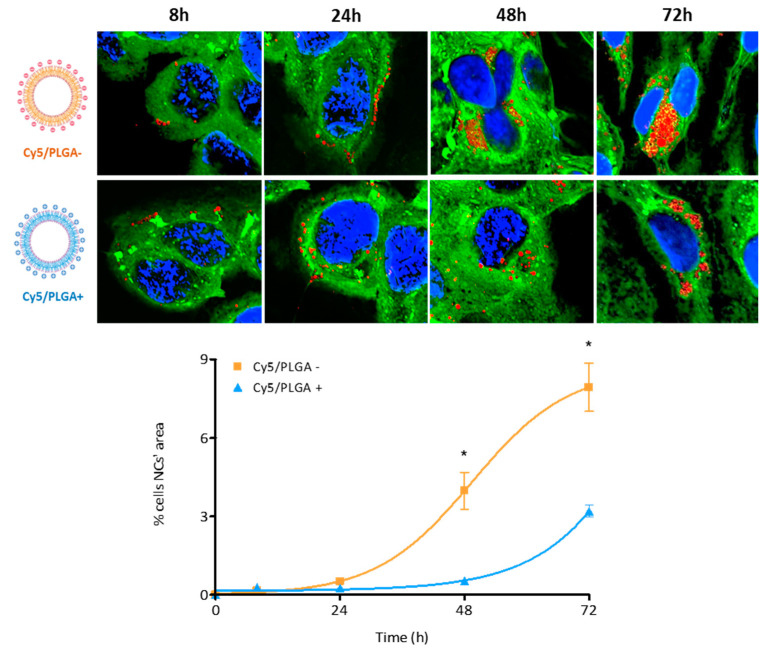
Confocal images of the intracellular localization of NCs in HPAEpiC cells: Representation of the percentage of the cellular area that PLGA NCs occupy. Both types of NCs increased their deposition within the cells over time. However, Cy5/PLGA- NCs filled a higher percentage of the cells’ area in comparison with Cy5/PLGA+ NCs, occupying 7.9% and 3.2% at 72 h, respectively. Nuclei are blue (DAPI), cells’ cytoplasm is green (Cell Mask), and NCs are red (Cy5); 63× magnification. Data are representative of 2 independent experiments (mean ± SEM); two-way ANOVA followed by Bonferroni’s multiple comparison test was used to evaluate significant differences; * *p* < 0.001 vs. 48 and 72 h Cy5/PLGA+.

A similar trend was observed for HPAEpiC cells (Figure 6). Although both types of NCs increased their presence inside the cells in a time-dependent manner, anionic NCs occupied a higher percentage of the total cell area, reaching ~8% at 72 h, while cationic NCs only filled 3.2% at the end of the treatment. This significant difference between anionic and cationic NCs (*p* < 0.001) may be due to alternative intracellular placement, as Harush-Frenkel et al. reported [37]. Moreover, it has also been observed that the surface charge of the NCs can be decisive in the pathway by which they are captured [38,39], directly affecting their localization within the cells and, consequently, the pH to which they will be exposed [40]. Further studies are needed to clarify the NCs’ intracellular disposition.

### 3.5. Nanocapsules’ Biodistribution and Cellular Uptake In Vivo

**Figure 7:**
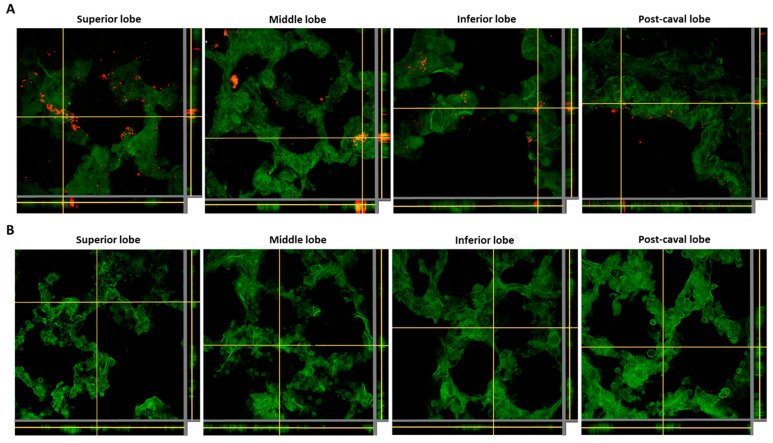
Z-stacking analysis with xz and yz orthogonal views of lung (superior, middle, inferior, and post-caval lobes) tissue slices of animals administered with Cy5/PLGA- NCs for 1 h through intratracheal instillation (**A**) or the intravenous route (**B**). Green: membranes stained with Cell Mask; red: NCs (Cy5); 63× magnification; average zoom = 3×.

**Figure 8:**
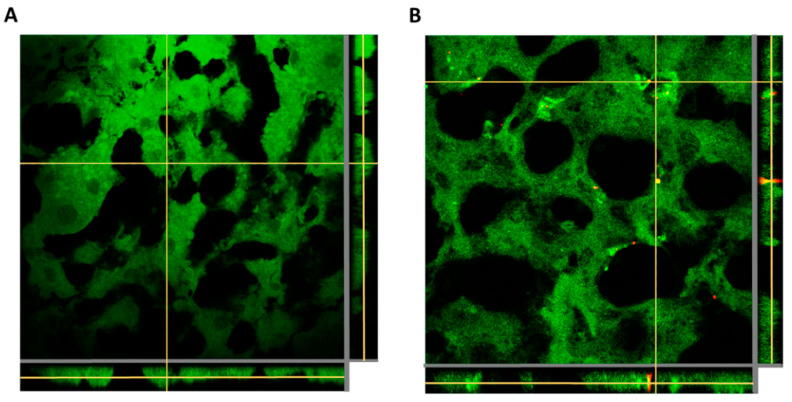
Z-stacking analysis with xz and yz orthogonal views of liver tissue slices of animals administered with Cy5/PLGA- NCs for 1 h through intratracheal instillation (**A**) or the intravenous route (**B**). Green: membranes stained with Cell Mask; red: NCs (Cy5); 63× magnification; average zoom = 3×.

Considering the faster cellular kinetics and higher uptake of the Cy5/PLGA- NCs for the two lung-related cell lines, this system was selected to study the NCs’ biodistribution and cellular internalization in vivo. Regarding NCs’ lung biodistribution analysis, the lung and liver tissues of rats administered with NCs through intratracheal instillation and intravenous administration for 1 h were analyzed. The red fluorescence belonging to the NCs was only detected in the lung tissue of the intratracheally administered animals. Significantly, this administration route facilitated the deposition of NCs in all lung lobules (Figure 7A), and avoided the NCs reaching the liver after 1 h of treatment (Figure 8A). In contrast, the intravenously administered animal presented retention of NCs in liver tissue (Figure 8B) without reaching the lungs (Figure 7B).

**Figure 9:**
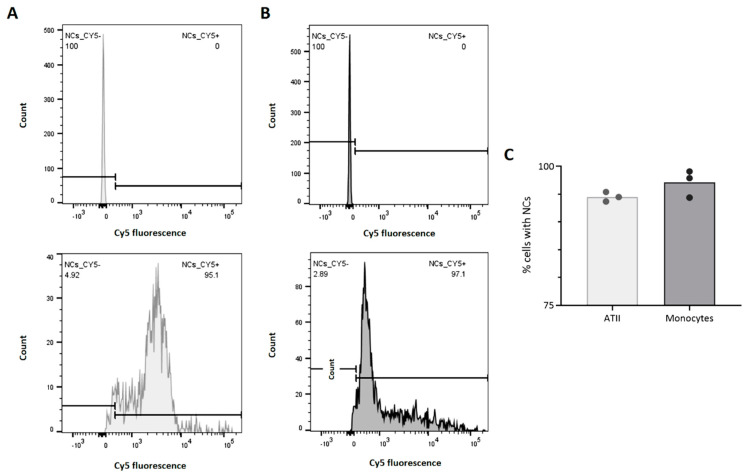
Representative flow cytometry histograms of ATII cells (**A**) and macrophages (**B**) from lung tissues of non-treated animals (upper graphs) and animals intratracheally administered with NCs for 24 h (lower graphs). Graphical representation of the percentages of ATII cells and macrophages that were positive for Cy5 fluorescence (**C**). Data shown as the mean ± SEM (n = 3).

Regarding the study of NCs’ internalization in vivo, an analysis of lung tissue was carried out by flow cytometry of those animals that were instilled with NCs for 24 h. By specifically labeling ATII cells and monocytes, we confirmed the good ability of NCs to be captured by the two main lung cell populations, which are essential for maintaining homeostasis and lung protection [27]. Specifically, we observed that 98% of monocytes and 96% of ATII cells contained NCs (Cy5 fluorescence) after 24 h of treatment (Figure 9). Moreover, these results reinforce the data obtained from the in vitro cellular uptake studies, which also showed that both cell types were able to reach an internalization rate of almost 100% after 24 h of treatment.

These preliminary preclinical studies allowed us to demonstrate that the modification of PLGA NCs with Cy5 offers a suitable tool to track these carriers in different lung tissue cell populations via both confocal microscopy and flow cytometry. Moreover, a significant advantage of using long-wavelength dyes, such as Cy5, is that they enable a distinctly high signal–noise ratio because of the low autofluorescence of biological specimens in the region of their spectra [41].

Together, the performed in vivo studies demonstrate that the newly developed PLGA carrier can be effectively and safely administered via the two administration routes, as none of the animals showed symptoms of discomfort, and the ones that received the NCs via pulmonary delivery were breathing normally. Furthermore, comparing the two delivery pathways reinforced that intratracheal administration is the most appropriate route in preclinical studies [20], as it allows precise control of the dose given to each animal, while effectively delivering the NCs to the lungs.

## 4. Conclusions

This study describes the synthesis and characterization of fluorescent PLGA nanocarriers with different surface charges as a promising drug delivery system for treating lung diseases. The in vitro studies showed that positively and negatively charged NCs were well preserved after cryo-storage for at least one month. The NCs are colloidally stable in all cell cultures and biologically relevant lung media studied, and did not exhibit cytotoxic effects—even at very high concentrations—on the two representative cell types present in the pulmonary environment. Remarkably, we observed a significant difference in cellular NCs’ uptake kinetics and deposition amounts between anionic and cationic NCs. The uptake of the anionic system was faster and in much higher amounts. This confirms that the surface charge plays a crucial role in NCs’ internalization, and probably in their destination within the cell. Further studies are needed to understand the intracellular pathways of those PLGA NCs in more detail, since this is essential in determining their degradation rate and content release. Regarding the studied cell types, slower NC kinetics of internalization by alveolar epithelial cells was established compared to macrophages. The in vivo studies demonstrated that these new carriers could be safely administered intratracheally and intravenously. Notably, they are promising as a pulmonary drug delivery system due to their excellent retention in lung tissue and their ability to be internalized by alveolar epithelial cells and macrophages—the principal targets of a pulmonary-delivered drug. Overall, the biocompatible PLGA nanocarriers developed in this study have great potential as pulmonary drug delivery systems, with a marked translational character that, in the future, will make them a valuable and versatile tool for the treatment of lung-injury-related diseases.

## Figures and Tables

**Table 1:** List of conjugated antibodies used in the flow cytometry analysis.

*Antibody*	*Fluorochrome*	*Source*	*Reference*	*Dilution*
*CD45*	Pacific Blue	BioLegend	202226	1:200
*CD11b*	PE-Cy7	BioLegend	201817	1:200
*His48*	FITC	eBioscience	15268119	1:200
*AP*	Alexa Fluor 546	Santa Cruz Biotechnology	sc-271431	1:50
